# The complete chloroplast genomes of *Polygonatum hunanense*, *P. verticillatum*, and *P. caulialatum* and their phylogenetic positions

**DOI:** 10.1080/23802359.2024.2357681

**Published:** 2024-06-07

**Authors:** Qing-Dong Ma, Hong-Jing Zhang, Yan-Ran Qi, Zheng-You Yin, Dong-Yang Yi, Si-Rong Yi

**Affiliations:** a School of Pharmacy, Chongqing Three Gorges Medical College, Chongqing, PR China; b Chongqing Key Laboratory of Development and Utilization of DaoDi Medicinal Materials in Three Gorges Reservoir Area, Chongqing, PR China; c Chongqing Engineering Research Centre of Antitumor Natural Drugs, Chongqing, PR China

**Keywords:** Chloroplast genome, *Polygonatum hunanense*, *Polygonatum kingianum* var. *grandifolium*, *Polygonatum verticillatum*, *Polygonatum caulialatum*

## Abstract

*Polygonatum hunanense* H.H. Liu & B.Z. Wang (2021) and *P. verticillatum* (L.) All. (1875) have been widely used as foods and as folk medicines in China and India, and *P. caulialatum* S. R. Yi (2021) has recently been described as a new medical plant in China. There is at present a lack of genome information regarding the species. Hence, this study reports the complete chloroplast genomes of the three species. The genomes of *P. hunanense*, *P. verticillatum*, and *P. caulialatum* were 155,583 bp, 155,650 bp, and 155,352 bp in length, respectively. They contained large single-copy (LSC) regions of 84,412 bp, 84,404 bp, and 84,285 bp, small single-copy (SSC) regions of 18,427 bp, 18,416 bp, and 18,463 bp, and a pair of inverted repeats of 26,372 bp, 26,415 bp, and 26,302 bp, respectively. The chloroplast genomes of *P. hunanense*, *P. verticillatum*, and *P. caulialatum* had 133 (103 unique) genes, consisting of 87 protein-coding genes, 38 ribosomal ribonucleic acid (RNA) genes, and eight transfer RNA genes, respectively. A maximum-likelihood phylogenetic tree showed that *P. kingianum* Coll. et Hemsl. var. *grandifolium* D.M. Liu & W.Z. Zeng (1991) was closer to *P. cyrtonema* Hua (1892) rather than to *P. kingianum* Coll. et Hemsl. (1890), further supporting its status as a unique species of the genus. Moreover, *P. verticillatum* was separated from the easily confused herb *P. cirrhifolium* (Wall.) Royle (1839), while *P. caulialatum* was closest to *P. humile* Fisch. ex Maxim. (1859). This research provides a foundation for further study of these herbs.

## Introduction

Polygonati rhizoma has been employed as a tonic and a traditional Chinese medicine for over 2000 years, being registered in the *Chinese Pharmacopoeia* as ‘Huang Jing’ and comprising *Polygonatum kingianum* Coll.et Hemsl. (1890), *P. sibiricum* Red. (1812), and *P. cyrtonema* Hua (1892) (Jiang et al. 2022). In addition, there are more than 10 species of *Polygonatum* that are cultivated, produced, and sold as medical plants in Chinese markets (Yang et al. 2020).

**Figure 1.**
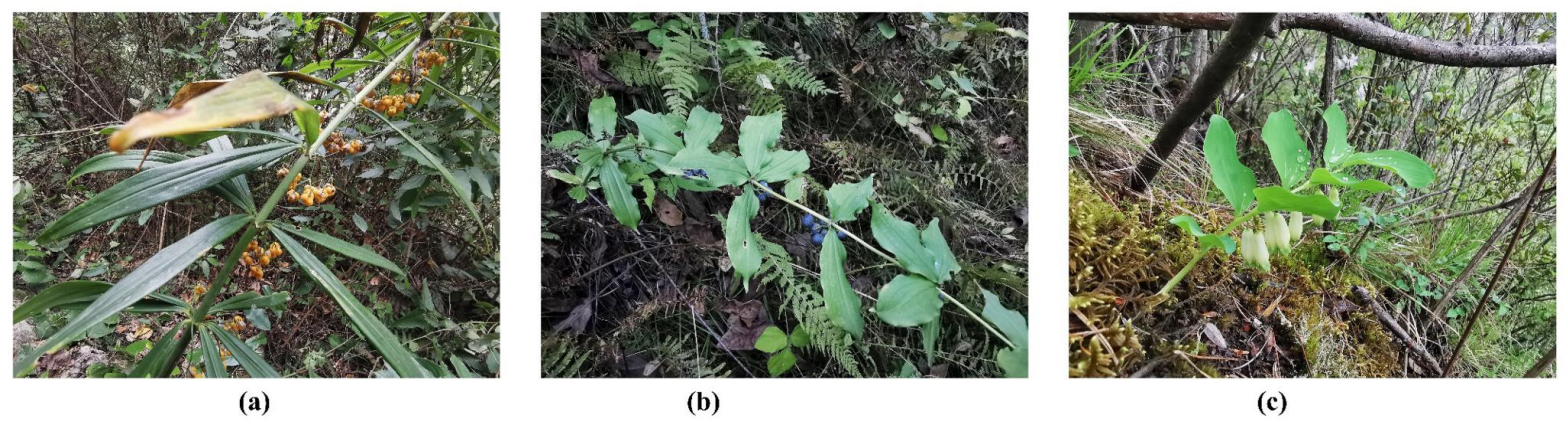
Photographs of *Polygonatum hunanense* (a), *P. verticillatum* (b), and *P. caulialatum* (c) taken by Si-Rong Yi at Wushan (N31°00′, E109°75′) and Chengkou (N31°85′, E109°12′; N32°05′, E108°83′, respectively), Chongqing, China. Core features: (a) rhizome ginger-like, 2–4 cm in diameter. Stem over 100 cm tall; leaves whorled, opposite, or alternate, elliptic to oblong-lanceolate, 8–25 cm long, 1.5–3.5 cm wide, apex cirrose to curved. Inflorescences with 2–10 flowers, pedicels 4–10 mm long. Perianth white, light yellow-green, or light purple, 6–9 mm long, cylindrical, slightly constricted in the middle. Berries spherical, yellow when ripe, 6–7 mm in diameter, with 10–20 seeds. (b) Rhizome, internode 2–3 cm long, thick at one end and thin at the other end. Stem 40–80 cm tall, with three leaves in whorls, elliptical to rectangular, 6–10 cm long, 2–3 cm wide, apex acute to acuminate. Inflorescence with 1 or 2–4 flowers, pedicels 1–2 cm long. Flowers pendulous, bracts small or absent. Perianth pale yellow or light purple, 8–12 mm long, lobes 2–3 mm long. Berries, red, 6–9 mm in diameter, 6–12-seeded. (c) Rhizome moniliform, 1.5–2.5 cm in diameter. Stem 40–80 cm tall, leaves 7–15, alternate, oblong, 8–15 cm long, 3.5–5 cm wide. Inflorescences axillary, 1–2-flowered, sparsely 3-flowered; pedicels 2.5–3.5 cm long. Corolla campanulate terete, 2.8–3.2 cm long, 8–10 mm in diameter, green-white. Berries subglobose, red, 8–15 mm in diameter, 3-loculed, 2–4 seeds per locule; seeds subglobose, 3.2–4 mm in diameter.

Among the above species, *P. kingianum* Collett et Hemsl. var. *grandifolium* D.M. Liu & W.Z. Zeng (1991) is the major medicinal variety cultivated in Sichuan and Chongqing as polygonati rhizoma due to its large size, sweet taste, and strong adaptability (Lu et al. 2019). However, the classification of *P. kingianum* var. *grandifolium* remains controversial; it was initially recorded as a variety of *P. kingianum* named ‘Daye Huangjing’ in the *Flora of Sichuan* in 1991 and as a synonym for *P. kingianum* in the *Flora of China* in 2000 (Shi et al. 2022). Based on morphological and molecular evidence, *P. kingianum* var. *grandifolium* had been proposed as a newly recognized synonym of *P. hunanense* H.H. Liu & B.Z. Wang (2021) (Figure 1(a)) as ‘Xiang Huangjing’ in Chinese (Liu et al. 2021; Xia et al. 2021), and it was more recently regarded as a species of *P. cyrtonema* (Ma et al. 2022). In the meantime, *P. verticillatum* (L.) All. (1875) (Figure 1(b)) has been widely used as a food and a folk medicine in China and India, including as a substitute for *P. kingianum* in Yunnan province of China, but it is among the least investigated species in the genus *Polygonatum* Mill. (Zhou et al. 2017; Sharma et al. 2021). Recently, another new medicinal plant in this genus, *P. caulialatum* S. R. Yi (2021) (Figure 1(c)), was discovered in Sichuan province by Si-Rong Yi from our team (Chen et al. 2021). Coincidentally, all three herbs are distributed in the mountains of northeastern Chongqing municipality in China, but little is known concerning their genomic information. Furthermore, DNA barcoding based on ITS/ITS2 sequences has had low identification efficiency toward interspecies relationships within the genus *Polygonatum* Mill. (Long et al. 2022; Zhang MY et al. 2023). To provide additional data for research and development of the herbs, in this study, we obtained the complete chloroplast genomes and analyzed the relationships between the three species.

## Materials and methods

The fresh leaves of *P. hunanense* were collected from Wushan, Chongqing, China (N31°00′, E109°75′), and the fresh leaves of *P. verticillatum* and *P. caulialatum* were collected from Chengkou, Chongqing, China (N31°85′, E109°12′; N32°05′, E108°83′, respectively). The specimens were deposited in the herbarium of Chongqing Three Gorges Medical College (*P. hunanense*: YSR0891, *P. verticillatum*: the specimen was planted in the herb garden of the same college, *P. caulialatum*: YSR1458; Chongqing, China; Si-Rong Yi; yisirong123@aliyun.com). After the total genomic DNA was extracted, high-throughput sequencing was performed with an Illumina HiSeq X Ten platform, and bioinformatic data for *P. hunanense*, *P. verticillatum*, and *P. caulialatum* were then assembled from the reference sequences of *P. hunanense* (MZ286311, Ji 2022a), *P. zanlanscianense* Pamp. (1915) (ON534059, Zhang DJ et al. 2023), and *P. caulialatum* (NC_068899, Hu et al. 2022), respectively (Hahn et al. 2013; Zhou et al. 2022). Genome maps, coverage-depth maps, and cis-/trans-splicing gene maps were constructed using the CPGview online tools (Liu et al. 2023).

To analyze the phylogenetic relationships among *P. hunanense*, *P. verticillatum*, and *P. caulialatum* within the genus *Polygonatum*, the chloroplast genome data of 29 species of *Polygonatum* were downloaded. These genome sequences, based on protein-coding genes, were aligned and processed by Geneious R11, and then imported to TOPALI v2.5 software and RAxML version 8.2.12 to construct a maximum-likelihood (ML) phylogenetic tree (Milne et al. 2009; Alexandros Stamatakis 2014).

## Results and discussion

**Figure 2.**
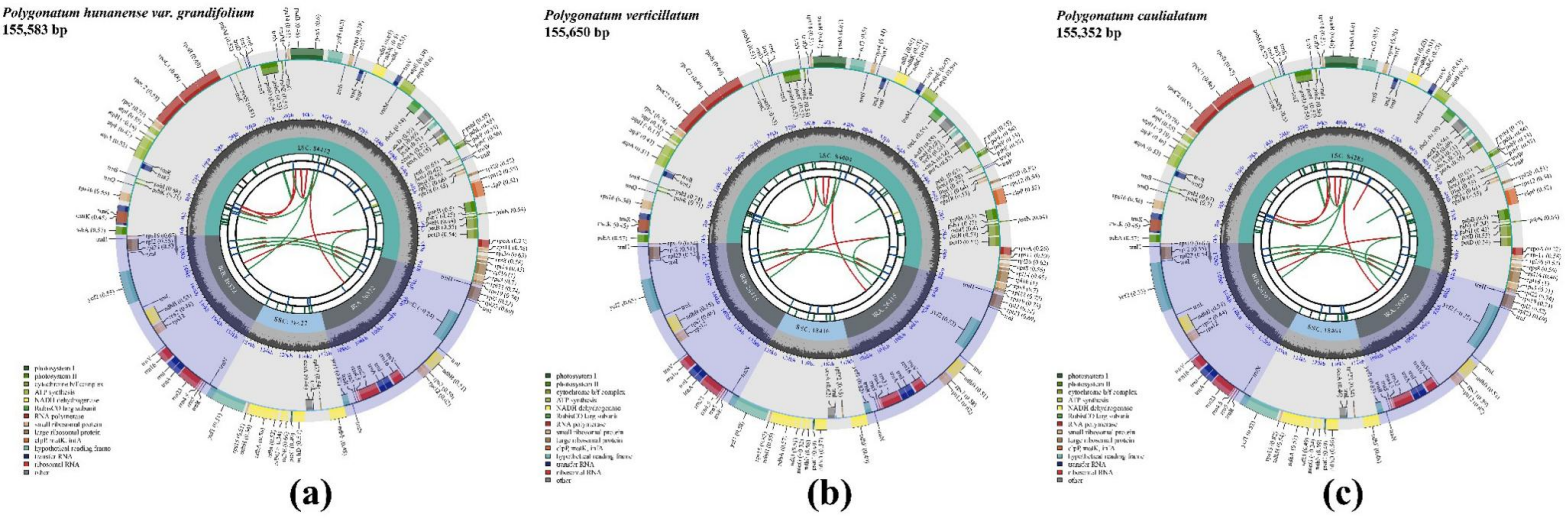
Chloroplast genome maps of *Polygonatum hunanense* (a), *P. verticillatum* (b), and *P. caulialatum* (c). The chloroplast genome structure consists of a typical tetrad circular molecule with a large single-copy (LSC) region, a small single-copy (SSC) region, and a pair of inverted repeats (IRa and IRb) that separate the LSC and SSC. The transcription direction of genes inside is clockwise, while the transcription direction of genes outside is counterclockwise. Genes are color-coded by their functional classification as shown in the lower-left corner.

Analysis of the depth of coverage of the complete chloroplast genomes and the annotation accuracy of several difficult genes suggested that the results for the complete chloroplast genomes of the three medical plants were trustworthy (Figures S1 and S2). Each group of raw reads of *P. hunanense*, *P. verticillatum*, and *P. caulialatum* indicated respective genome lengths of 155,583 bp, 155,650 bp, and 155,352 bp, comprising a typical tetrad circular molecule with a large single-copy (LSC) region, a small single-copy (SSC) region, and a pair of inverted repeats (IRs) that separated the LSC and SSC (Figure 2). The LSC regions of *P. hunanense* (GenBank accession no. OR386975), *P. verticillatum* (OR255921), and *P. caulialatum* (OR386974) were 84,412 bp, 84,404 bp, and 84,285 bp, respectively, in length; while their SSC regions were 18,427 bp, 18,416 bp, and 18,463 bp, respectively, in length, and the sizes of their IR regions ranged from 26,302 bp to 26,415 bp (Table S1). A total of 133 genes (103 different genes), including 87 protein-coding genes, 38 transfer ribonucleic acid (RNA) genes, and eight ribosomal RNA genes, were detected in the complete chloroplast genomes of the three herbs. The total guanine–cytosine (GC) content of the complete chloroplast genomes was 37.7%. The IR regions contained the highest GC content at 42.9%–43.0%, followed by the LSC regions at 35.7%; in contrast, the SSC regions had the lowest GC content at 31.5%–31.6%. This indicated an unbalanced distribution of the GC content among the regions.

The annotation results also showed that all functional genes comprised three categories: photosynthesis, self-replication, and other genes (Table S2). Among the genes, nine different genes (*atpF*, *ndhA*, *ndhB*, *petB*, *petD*, *rpl2*, *rpl16*, *rpoC1*, and *rpsl6*) contained one intron, and the *clpP* gene contained two introns. Meanwhile, seven genes (*ndhB*, *rpl2*, *rpl23*, *rps7*, *rps19*, *ycf1*, and *ycf2*) were duplicated, and the *rps12* gene was triplicated. Furthermore, 44, 45, and 49 simple sequence repeats in *P. hunanense*, *P. verticillatum*, and *P. caulialatum* were identified, respectively, consisting of mononucleotides, dinucleotides, and trinucleotides (Table S3). The polymers of A/T dominated in simple sequence repeats, ranging from 85.71% to 88.89%, indicating a remarkable base preference. Moreover, the number of codons in *P. hunanense*, *P. verticillatum*, and *P. caulialatum* was 20,297, 20,301, and 21,031, respectively (Table S4). Leucine was the most abundant amino acid, accounting for 10.2%–10.3% of the total codons; in contrast, cysteine comprised the least number of codons. This indicated a preference in the codons.

**Figure 3.**
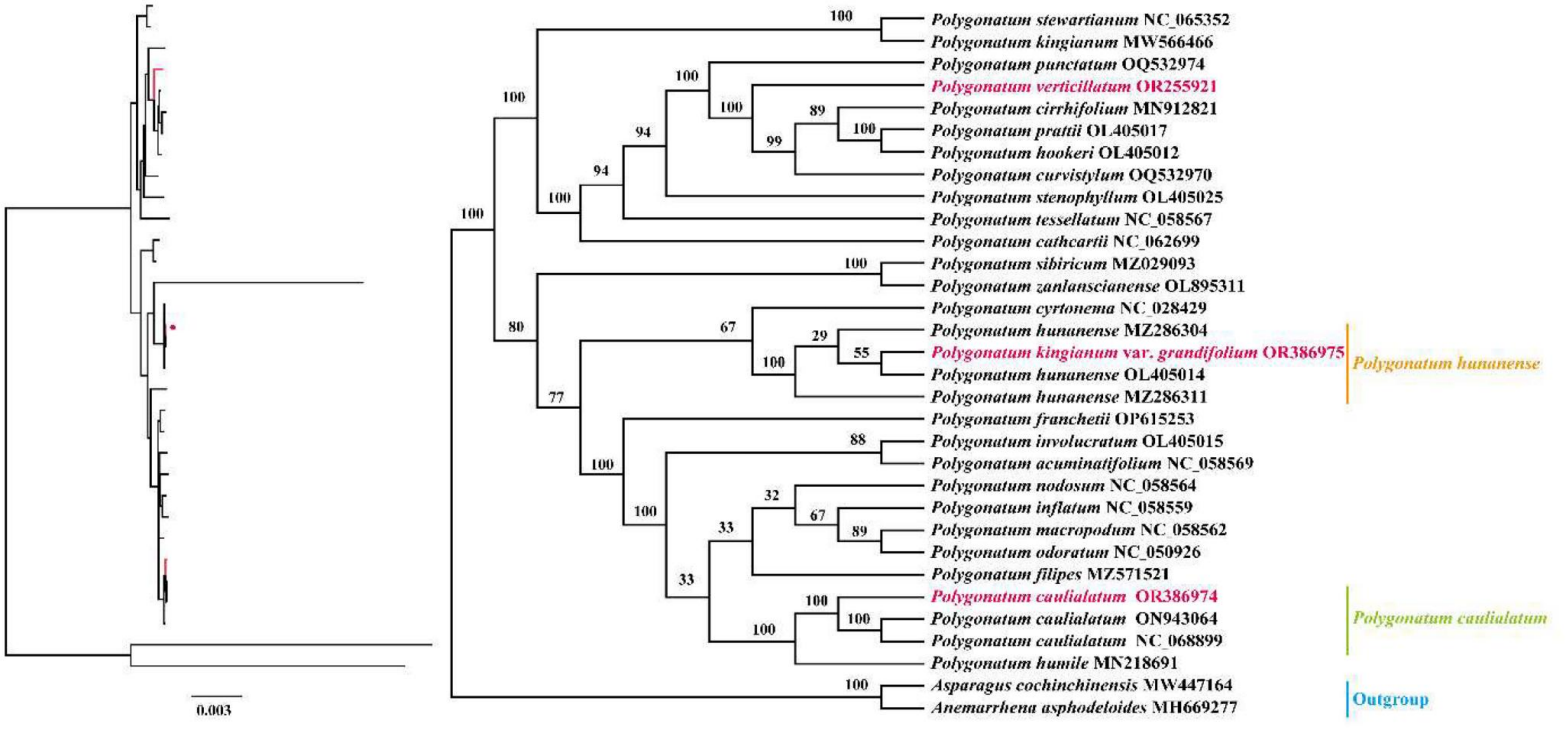
Maximum-likelihood (ML) phylogenetic tree of *Polygonatum hunanense*, *P. verticillatum*, *P. caulialatum*, and 29 *Polygonatum* species. The branches of the ML tree were supported using 1000 bootstrap replicates. The following sequences were used: *P. stewartianum* Diels (1912) NC_065352 (Wang J *et al.* 2022), *P. kingianum* MW566466 (Guo *et al.* 2022), *P. punctatum* Royle ex Kunth (1850) OQ532974 (Ji 2023a), *P. cirrhifolium* (Wall.) Royle (1839) MN912821 (Wu and Xiao 2022), *P. prattii* Baker (1892) OL405017 (Wang J *et al.* 2022), *P. hookeri* Baker (1875) OL405012 (Wang J *et al.* 2022), *P. curvistylum* Hua (1892) OQ532970 (Ji 2023b), *P. stenophyllum* Maxim. (1859) OL405025 (Wang J *et al* 2022), *P. tessellatum* Wang et Tang (1936) NC_058567 (Xia *et al.* 2022), *P. cathcartii* Baker (1875) NC_062699 (Zhao 2023), *P. sibiricum* MZ029093 (Wang J and Duan 2021), *P. zanlanscianense* OL895311 (Liu *et al.* 2022), *P. cyrtonema* NC_028429 (Wang S *et al.* 2023), *P. hunanense* MZ286304 (Ji 2022b), OL405014 (Wang J *et al.* 2022), *P. franchetii* Hua (1892) OP615253 (Wang L 2022), *P. involucratum* (Franch.et Sav.) Maxim. (1883) OL405015 (Wang J *et al.* 2022), *P. acuminatifolium* Kom. (1916) NC_058569 (Xia *et al.* 2022), *P. nodosum* Hua (1892) NC_058564 (Xia *et al.* 2022), *P. inflatum* Kom. (1901) NC_058559 (Xia *et al.* 2022), *P. macropodum* Turcz. (1832) NC_058562 (Xia *et al.* 2022), *P. odoratum* (Mill.) Druce (1906) NC_050926 (Du *et al.* 2020), *P. filipes* Merr. (1959) MZ571521 (Yan and Cheng 2022), *P. caulialatum* ON943064 (Hu *et al.* 2022), *P. humile* MN218691 (Lee *et al.* 2019), *Asparagus cochinchinensis* (Lour.) Merr. (1919) MW447164 (Sheng W 2021), and *Anemarrhena asphodeloides* Bunge (1831) MH669277 (Li *et al.* 2019).

Chloroplast genomes play an important role in phylogenetic analysis and species identification (Wu et al. 2020). The ML phylogenetic tree (Figure 3) showed that *P. hunanense* was close to *P. cyrtonema* rather than to *P. kingianum*, further supporting it as a unique species in the genus. This was consistent with the conclusions of a previous study (Guo et al. 2022). Moreover, *P. verticillatum* was separated from the easily confused herb *P. cirrhifolium*, while *P. caulialatum* was closest to *P. humile*.

## Conclusions

In this study, the complete chloroplast genomes of *P. hunanense*, *P. verticillatum*, and *P. caulialatum* were sequenced and annotated into a typical tetrameric structure that was similar to those of other *Polygonatum* species. The ML phylogenetic tree based on protein-coding genes in the chloroplast genomes suggested distinguishing between *P. kingianum* var. *grandifolium* and *P. kingianum*. The chloroplast genomes of the three herbs will provide information for identification and classification of the species that can be used to explore the evolution of the genus *Polygonatum*.

## Data Availability

The genome sequence data that support the findings of this research are openly available in GenBank of NCBI at https://www.ncbi.nlm.nih.gov/ under the accession nos. OR386975 (*P. hunanense*), OR255921 (*P. verticillatum*), and OR386974 (*P. caulialatum*). The associated BioProject, BioSample, and SRA numbers are PRJNA1020603; SAMN37526446 (*P. hunanense*), SAMN37526444 (*P. verticillatum*), and SAMN37526445 (*P. caulialatum*); SRR26159564 (*P. hunanense*), SRR26159566 (*P. verticillatum*), and SRR26159565 (*P. caulialatum*).

## References

[CIT0001] Chen HY, Huang Y, Zhou XX, Yi SR. 2021. *Polygonatum caulialatum*, a new species of medicinal plant of *Polygonatum* (Asparagaceae) from Sichuan, China. Phytotaxa. 513(1):55–61. doi:10.11646/phytotaxa.513.1.3.

[CIT0002] Du ZF, Qian J, Jiang Y, Duan BZ. 2020. The complete chloroplast genome of *Polygonatum odoratum* (Mill.) Druce and its phylogenetic analysis. Mitochondrial DNA B. 5(2):1601–1602. doi:10.1080/23802359.2020.1745101.

[CIT0003] Guo XR, Shi NX, Xie PX, Zhang GF, Liu HY, Ji YH. 2022. Plastome sequencing for accurate and effective authentication of *Polygonatum kingianum* (Asparagaceae). Ind Crops Prod. 184:115056. doi:10.1016/J.INDCROP.2022.115056.

[CIT0004] Hahn C, Bachmann L, Chevreux B. 2013. Reconstructing mitochondrial genomes directly from genomic next-generation sequencing reads—a baiting and iterative mapping approach. Nucleic Acids Res. 41(13):e129. doi:10.1093/nar/gkt371. 23661685 PMC3711436

[CIT0005] Hu YF, Liu YF, Ali M, Wu W, Li XL, Chen LS, Shao JW. 2022. *Polygonatum praecox* (Asparagaceae), a new species from mid-eastern China revealed by morphological and molecular evidence. PhytoKeys. 211:125–138. doi:10.3897/phytokeys.211.90456. 36760726 PMC9878575

[CIT0006] Ji Y. 2022a. *Polygonatum hunanense* chloroplast, complete genome. [updated 2022 Jul 4; accessed 2023 Oct 12]. https://www.ncbi.nlm.nih.gov/nuccore/MZ286311.1/.

[CIT0007] Ji Y. 2022b. *Polygonatum hunanense* chloroplast, complete genome. [updated 2022 Jul 4; accessed 2024 Jan 10]. https://www.ncbi.nlm.nih.gov/nuccore/MZ286304.

[CIT0008] Ji Y. 2023a. *Polygonatum punctatum* voucher 16CS11940 chloroplast, complete genome. [updated 2023 Apr 2; accessed 2023 Oct 12]. https://www.ncbi.nlm.nih.gov/nuccore/OQ532974.1/.

[CIT0009] Ji Y. 2023b. *Polygonatum curvistylum* voucher Ji YH 2020333 chloroplast, complete genome. [updated 2023 Apr 2; accessed 2023 Oct 12]. https://www.ncbi.nlm.nih.gov/nuccore/OQ532970.

[CIT0010] Jiang W, Li YP, Chen JD, Tao ZM. 2022. Genetic diversity analysis of Polygonati Rhizoma based on ISSR and SRAP molecular markers. Chin Tradit Herb Drugs. 53(21):6865–6873. doi:10.7501/j.issn.0253-2670.2022.21.025.

[CIT0011] Lee SY, Zou YL, Liao WB, Fan Q. 2019. The complete chloroplast genome of a traditional medicinal and food plant, *Polygonatum humile* (Asparagaceae, Asparagales). Mitochondrial DNA B Resour. 4(2):3184–3185. doi:10.1080/23802359.2019.1666044. 33365910 PMC7706532

[CIT0012] Li F, Li C, Tian EW, Chen AM, Ye HT, Shu YQ, Chao Z. 2019. The complete chloroplast genome sequence of *Anemarrhena asphodeloides* Bunge. Mitochondrial DNA B. 4(1):441–442. doi:10.1080/23802359.2018.1545542.

[CIT0013] Liu HH, Ma YS, Wang BZ, Jie HD, Xiang SJ, Yang JN, Jie YC. 2021. Morphological and molecular character on a new species of *Polygonatum hunanens* from Hunan, China. Crop Res. 35(1):88–94. doi:10.16848/j.cnki.issn.1001-5280.2021.01.15.

[CIT0014] Liu SY, Ni Y, Li JL, Zhang XY, Yang H, Chen HM, Liu C. 2023. CPGView: a package for visualizing detailed chloroplast genome structures. Mol Ecol Resour. 23(3):694–704. doi:10.1111/1755-0998.13729. 36587992

[CIT0015] Liu Y, Jin C, Zhang X, Zhou Y, Hu Y. 2022. *Polygonatum zanlanscianense* chloroplast, complete genome. [updated 2022 Oct 17; accessed 2023 Oct 12]. https://www.ncbi.nlm.nih.gov/nuccore/OL895311.1/.

[CIT0016] Long BH, Jiang XH, Song R, Li SH, Xiao LQ, Yi ZL, She CW. 2022. Application of DNA barcodes in identification and genetic diversity analysis of medicinal plants of the genus *Polygonatum*. Plant Sci J. 40(4):533–543. doi:10.11913/PSJ.2095-0837.2022.40533.

[CIT0017] Lu J, Zhang CH, Wu Y. 2019. Investigating tissue culture technique of a *Polygonatum kingianum* variety distributed in *Sichuan*. Chin J Appl Environ Biol. 25(5):1222–1227. doi:10.19675/j.cnki.1006-687x.2018.11043.

[CIT0018] Ma CD, Chang H, Yang YC, Wang EH, Zhan ZL. 2022. Herbal textual research on Polygonati Rhizoma in famous classical formulas. Chin J Exp Tradit Med Formul. 28(10):193–206. doi:10.13422/j.cnki.syfjx.20211865.

[CIT0019] Milne I, Lindner D, Bayer M, Husmeier D, McGuire G, Marshall DF, Wright F. 2009. TOPALi v2: a rich graphical interface for evolutionary analyses of multiple alignments on HPC clusters and multi-core desktops. Bioinformatics. 25(1):126–127. doi:10.1093/bioinformatics/btn575. 18984599 PMC2638937

[CIT0020] Sharma S, Joshi R, Kumar D. 2021. Metabolomics insights and bioprospection of *Polygonatum verticillatum*: an important dietary medicinal herb of alpine Himalaya. Food Res Int. 148:110619. doi:10.1016/j.foodres.2021.110619. 34507763

[CIT0021] Sheng W. 2021. *Asparagus cochinchinensis* chloroplast, complete genome. [updated 2021 Jun 30; accessed 2024 Jan 10]. https://www.ncbi.nlm.nih.gov/nuccore/MW447164.

[CIT0022] Shi Y, Yang TG, Yang MS, Yu M, Zhang XF. 2022. Polygonati Rhizoma: a crop with potential of being consumed as food and medicine. Zhongguo Zhong Yao Za Zhi. 47(4):1132–1135. doi:10.19540/j.cnki.cjcmm.20211105.101. 35285215

[CIT0023] Stamatakis A. 2014. RAxML version 8: a tool for phylogenetic analysis and post-analysis of large phylogenies. Bioinformatics. 30(9):1312–1313. doi:10.1093/bioinfor-matics/btu033. 24451623 PMC3998144

[CIT0024] Wang J, Duan B. 2021. *Polygonatum sibiricum* chloroplast, complete genome. [updated 2021 Aug 7; accessed 2023 Oct 12]. https://www.ncbi.nlm.nih.gov/nuccore/MZ029093.

[CIT0025] Wang J, Qian J, Jiang Y, Chen XC, Zheng BJ, Chen SL, Yang FJ, Xu ZC, Duan BZ. 2022. Comparative analysis of chloroplast genome and new insights into phylogenetic relationships of *Polygonatum* and tribe polygonateae. Front Plant Sci. 13:882189. doi:10.3389/FPLS.2022.882189. 35812916 PMC9263837

[CIT0026] Wang L. 2022. *Polygonatum franchetii* chloroplast, complete genome. [updated 2022 Nov 13; accessed 2023 Oct 12]. https://www.ncbi.nlm.nih.gov/nuccore/OP615253.1/.

[CIT0027] Wang S, Dang K, Niu J, Wang S, Wang L. 2023. *Polygonatum cyrtonema* chloroplast, complete genome. [updated 2023 Apr 3; accessed 2023 Oct 12]. https://www.ncbi.nlm.nih.gov/nuccore/NC_028429.1/.

[CIT0028] Wu LW, Nie LP, Xu ZC, Li P, Wang Y, He CN, Song JY, Yao H. 2020. Comparative and phylogenetic analysis of the complete chloroplast genomes of three *Paeonia* section Moutan species (Paeoniaceae). Front Genet. 11:980. doi:10.3389/fgene.2020.00980. 33193580 PMC7533573

[CIT0029] Wu T, Xiao L. 2022. *Polygonatum cirrhifolium* chloroplast, complete genome. [updated 2022 Apr 18; accessed 2023 Oct 12]. https://www.ncbi.nlm.nih.gov/nuccore/MN912821.

[CIT0030] Xia MQ, Liu Y, Liu JJ, Chen DH, Shi Y, Bai ZC, Xiao Y, Peng C, Si JP, Li P, et al. 2021. A new synonym of *Polygonatum* in China, based on morphological and molecular evidence. PhytoKeys. 175:137–149. doi:10.3897/phytokeys.175.63383. 34475796 PMC8390792

[CIT0031] Xia MQ, Liu Y, Liu JJ, Chen DH, Shi Y, Chen ZX, Chen DR, Jin RF, Chen HL, Zhu SS, et al. 2022. Out of the Himalaya-Hengduan Mountains: phylogenomics, biogeography and diversification of *Polygonatum* Mill. (Asparagaceae) in the Northern Hemisphere. Mol Phylogenet Evol. 169:107431. doi:10.1016/j.ympev.2022.107431. 35131418

[CIT0032] Yan M, Cheng R. 2022. *Polygonatum filipes* chloroplast, complete genome. [updated 2022 Aug 6; accessed 2023 Oct 12]. https://www.ncbi.nlm.nih.gov/nuccore/MZ571521.1/.

[CIT0033] Yang ZY, Yang K, Zhu XX, Liu XM, Hu Q, Chen Y. 2020. Development status and industry development analysis of Polygonati Rhizoma health-care food. J Tradit Chin Med Univ Hunan. 40(7):853–859. doi:10.3969/j.issn.1674-070X.2020.07.014.

[CIT0034] Zhang DJ, Ren J, Jiang H, Wanga VO, Dong X, Hu GW. 2023. Comparative and phylogenetic analysis of the complete chloroplast genomes of six *Polygonatum* species (Asparagaceae). Sci Rep. 13(1):7237. doi:10.1038/s41598-023-34083-1. 37142659 PMC10160070

[CIT0035] Zhang MY, Li YM, Cheng WP, Gao J, Yan YG, Yang L, Hu JH, Zhang G. 2023. Molecular authentication of medicinal *Polygonatum* species utilizing the universal DNA barcode sequences. Chin Tradit Herb Drugs. 54(1):235–244. doi:10.7501/j.issn.0253-2670.2023.01.025.

[CIT0036] Zhao L. 2023. *Polygonatum cathcartii* plastid, complete genome. [updated 2023 Apr 3; accessed 2023 Oct 12]. https://www.ncbi.nlm.nih.gov/nuccore/NC_062699.1/.

[CIT0037] Zhou JC, Zhang JZ, Qiu YJ, Fan CZ. 2022. The complete chloroplast genome sequence of *Viola kunawarensis* Royle, a precious Uygur medicinal material. Mitochondrial DNA B Resour. 7(9):1704–1706. doi:10.1080/23802359.2022.2122882. 36188669 PMC9518287

[CIT0038] Zhou PJ, Li XF, Fu DH, Pu XY, Zhu GQ. 2017. Comparative identification of *Polygonatum kingianum* Coll. et Hemsley and its adulterants *Polygonatum verticillatum* (L.) All. J Guangzhou Univ Tradit Chin Med. 34(4):587–591. doi:10.13359/j.cnki.gzxbtcm.2017.04.026.

